# Imaging of Orthotopic Glioblastoma Xenografts in Mice Using a Clinical CT Scanner: Comparison with Micro-CT and Histology

**DOI:** 10.1371/journal.pone.0165994

**Published:** 2016-11-09

**Authors:** Stefanie Kirschner, Bettina Mürle, Manuela Felix, Anna Arns, Christoph Groden, Frederik Wenz, Andreas Hug, Gerhard Glatting, Martin Kramer, Frank A. Giordano, Marc A. Brockmann

**Affiliations:** 1 Department of Neuroradiology, University Medical Center Mannheim, Medical Faculty Mannheim, Heidelberg University, 68167, Mannheim, Germany; 2 Medical Radiation Physics/Radiation Protection, Department of Radiation Oncology, University Medical Center Mannheim, Medical Faculty Mannheim, Heidelberg University, 68167, Mannheim, Germany; 3 Department of Radiation Oncology, University Medical Center Mannheim, Medical Faculty Mannheim, Heidelberg University, 68167, Mannheim, Germany; 4 Spinal Cord Injury Center, University Hospital Heidelberg, Schlierbacher Landstr. 200a, 69118, Heidelberg, Germany; 5 Department of Veterinary Clinical Sciences, Small Animal Clinic, Justus-Liebig-University, 35392, Giessen, Germany; 6 Department of Neuroradiology, University Medical Center of the Johannes Gutenberg University Mainz, 55131, Mainz, Germany; Fraunhofer Research Institution of Marine Biotechnology, GERMANY

## Abstract

**Purpose:**

There is an increasing need for small animal in vivo imaging in murine orthotopic glioma models. Because dedicated small animal scanners are not available ubiquitously, the applicability of a clinical CT scanner for visualization and measurement of intracerebrally growing glioma xenografts in living mice was validated.

**Materials and Methods:**

2.5x10^6^ U87MG cells were orthotopically implanted in NOD/SCID/ᵞc^-/-^ mice (n = 9). Mice underwent contrast-enhanced (300 μl Iomeprol i.v.) imaging using a micro-CT (80 kV, 75 μAs, 360° rotation, 1,000 projections, scan time 33 s, resolution 40 x 40 x 53 μm) and a clinical CT scanner (4-row multislice detector; 120 kV, 150 mAs, slice thickness 0.5 mm, feed rotation 0.5 mm, resolution 98 x 98 x 500 μm). Mice were sacrificed and the brain was worked up histologically. In all modalities tumor volume was measured by two independent readers. Contrast-to-noise ratio (CNR) and Signal-to-noise ratio (SNR) were measured from reconstructed CT-scans (0.5 mm slice thickness; n = 18).

**Results:**

Tumor volumes (mean±SD mm^3^) were similar between both CT-modalities (micro-CT: 19.8±19.0, clinical CT: 19.8±18.8; Wilcoxon signed-rank test p = 0.813). Moreover, between reader analyses for each modality showed excellent agreement as demonstrated by correlation analysis (Spearman-Rho >0.9; p<0.01 for all correlations). Histologically measured tumor volumes (11.0±11.2) were significantly smaller due to shrinkage artifacts (p<0.05). CNR and SNR were 2.1±1.0 and 1.1±0.04 for micro-CT and 23.1±24.0 and 1.9±0.7 for the clinical CTscanner, respectively.

**Conclusion:**

Clinical CT scanners may reliably be used for *in vivo* imaging and volumetric analysis of brain tumor growth in mice.

## Introduction

Glioblastoma multiforme (GBM) is the most common primary brain tumor in adults with a poor prognosis. Life expectancy after diagnosis does usually not exceed two years [1]. Despite aggressive therapy including neurosurgical resection, radiotherapy, and chemotherapy, the mortality is 90–95%. In order to improve diagnosis and treatment of GBM, rodent models (mainly mice and rats) are being used for in vivo research. Even in small rodents like mice, noninvasive imaging techniques have been established to monitor disease development, progression and treatment effects in brain tumor models. Dedicated small animal magnetic resonance (MR) scanners [2–5], micro-computed tomography (micro-CT) [6–10], positron emission tomography/single-photon emission computed tomography (PET/SPECT) [2, 7, 9, 10], and bioluminescence imaging (BLI) [2, 4, 5, 11, 12] have been used for imaging of glioma growth in living mice. As these modalities are not ubiquitously available, clinical MR scanners have also been succesfully used for imaging of murine brain tumors [13, 14].

Compared to MRI, PET or SPECT, CT as a stand-alone approach is limited by an unfavorable soft tissue contrast, which might hamper sensitive detection of intracerebral tumors. Nevertheless, micro-CT has been successfully used for the evaluation of anatomical structures (e. g. vascular imaging, virtual colonoscopy) and pathological processes (e.g. intrahepatic, intrasplenic and intracerebral tumor growth) in animals as small as mice [6, 15–23]. Clinical scanners to some point are an alternative to dedicated small animal scanners as they are ubiquitously available and allow fast, easy and cost effective visualization of anatomical structures and some disease processes [24–26]. Whether clinical CT-scanners may be used for in vivo imaging of orthotopically growing brain tumors in mice has not been investigated yet.

Hence, this study was performed to evaluate the value of a clinical CT scanner for i.) the diagnosis of orthotopically growing brain tumors in mice *in vivo* and ii.) exploring in how far treatment effects on tumor morphology can be visualized.

## Material and Methods

### Cell culture

The human glioblastoma cell line U87MG (ATCC, Manassas, VA, USA) was grown in DMEM with 10% FBS (FBS; Biochrom AG, Berlin Germany) under 5% CO_2_ at 37°C. 24h before transplantation, medium was replaced by fresh DMEM with 10% FBS (FBS; Biochrom AG, Berlin Germany). Prior to transplantation subconfluent cultures of cells were washed twice with PBS (Biochrom AG, Berlin Germany) and trypsinized. Cells were counted and harvested by centrifugation. The cell pellet was resuspended in PBS at a concentration of 1 x 10^6^ cells/μl.

### Animals

9 to 12 week old male and female NOD/SCID gamma chain knock out (NSG, The Jackson Laboratory, USA) were used. All animals were housed in individually ventilated cages with controlled temperature (75±1°F), humidity (45–50%), room ventilation (10–15 air changes per hour), and a lighting cycle of 12 h light/dark. During the experimental period all mice had free access to a standard autoclaved research rodent diet (ssniff Spezialdiäten GmbH, Germany) and to autoclaved tap water.

### Implantation of glioma xenografts

All experiments were carried out after receiving the local ethics committee approval (Regierungspräsidium Karlsruhe, Baden-Württemberg, Germany). Institutional guidelines for animal welfare and experimental conduct were followed. All mice (n = 9) were anesthesized by subcutaneous injection with a mixture of medetomidine (0.5 mg/kg), midazolam (5 mg/kg) and fentanyl (0.05 mg/kg). Implantation of tumor cells was performed as described recently [6, 27]. Prior to surgery the animals received a subcutaneous injection of sodium chloride (10 ml/kg) to prevent dehydration. After reaching the surgical tolerance stage the head of the mouse was positioned in a stereotactic frame (TSE Systems, Bad Homburg, Germany) under an operating microscope and an ophthalmic ointment was applied. The depilated (with common hair removal cream) scalp was desinfected with povidone solution and a linear skin incision was made starting in the midline between the eyes and ending in the midline dorsal to the bregma. For intracerebral tumor cell injection, a 0.5 mm burr hole was drilled at 25,000 rpm (Marathon Escort 3, Saeyang, China) with a 0.5 mm rose-head burr (Gebr. Brasseler GmbH & Co. KG, Lemgo, Germany) 1 mm anterior to the bregma and 3 mm lateral to the midline. To inject 2.5 μl of the U87 MG cell suspension (10^6^/μl) a 25 μl gastight syringe (Neuros Syringe, Hamilton) with a 33 G blunt needle was used. To ensure targeted administration with reprodicible penetration depth, a depth stop was attached at 3 mm. The injection was carried out slowly over a period of 10 min to reduce the risk of reflux of the cell suspension. When the injection volume was completely administered the neddle was kept in place for another minute and then gradually retracted over a period of 3 min. Finally, the burr hole was sealed with bone wax and the scalp was closed with a tissue adhesive (Surgibond^®^). The total time for stereotactic implantation ranged between 20 and 30 min per mouse. After implantation, anesthesia was antagonized by subcutaneous injection of flumazenil (0.5 mg/kg), naloxon (1.2 mg/kg) and atipamezol (2.5 mg/kg) to accelerate recovery from intervention. For postoperative pain management all animals were treated with metamizole (Novalgin, 200 mg/kg) via drinking water on three consecutive days. At the end of the study animals were sacrificed by cervical dislocation under the same general anesthesia as mentioned above. Signs of neurologic deficits and/or loss of more than 20% of body weight were defined as latest humane endpoints. Two animals met the requirements for humane endpoints and were subsequently sacrificed. No animals died spontaneously.

### In vivo micro-CT imaging

A customized micro-CT (Y.Fox, Yxlon, Garbsen, Germany) equipped with an open multifocus x-ray tube and a 14-bit direct amorphous silicon flat panel detector (Varian PaxScan 2520 D/CL; Varian, Palo Alto, CA, USA) in which the object is rotated around its longitudinal axis in the cone beam was used as described previously [17, 19, 28]. Under general anesthesia directly before image acquisition, 300 μl of a prewarmed iodine-based contrast agent (Iomeprol; Imeron^®^ 300, Bracco Imaging Group, Konstanz, Germany) were injected over 40 s via the lateral tail vein. For injection a standard 1 ml syringe with a 30 G needle was used. For micro-CT imaging, mice were fixed in a custom-made acrylic cradle that was mounted onto the three-jaw drill chuck of the rotational axis of the micro-CT. The animal was held in the cradle during the rotation by adhesive tape circled around thorax and abdomen. The head was carefully secured with ear bars and an incisor bar. The source-object and object-detector distances were adjusted so that the head filled the field of view (FoV). Scans were performed 3–6 min after i.v. administration of contrast agent. Imaging was performed using the following scan parameters: tube voltage 80 kV; (current 75 μA), 360° rotation within 33 s scan time and continuous image acquisition at 30 frames per second resulting in 1,000 projections. To reduce skin doses, a 0.5 mm aluminium filter was used [29, 30]. Raw-data were reconstructed using a filtered back projection algorithm with a matrix of 512 x 512 x 512 (Reconstruction Studio, Tera Recon) [17, 18, 31, 32] and a smooth band-pass filter method, which we evaluated to provide the best soft tissue contrast. In-plane resolution was 40x40 μm. The resulting digital imaging an communications in medicine (DICOM) data were analysed by two readers using OsiriX imaging software (OsiriX v. 5.0.2, 64-bit). Tumor volumetry was performed by contouring contrast-enhancing regions of interest (ROIs) in coronal sections. Subsequently the volume of interest (VOI) was ascertained by applying a wrapping calculation to the previously measured regions of interest [33, 34].

### In vivo clinical CT imaging

CT scans using a clinical CT scanner (Somatom Volume Zoom; Siemens Medical Systems, Erlangen, Germany) were performed 6h after micro-CT scanning. Animals were prepared as mentioned in the micro-CT section, including the administration of the same dose of contrast agent. The scan protocol was as follows: tube voltage 120 kV, 150 mAs, slice thickness 0.5 cm, rotation time 0.75 s, feed rotation 0.5 mm. Images were reconstructed with a matrix of 512 x 512 pixels and a slice thickness of 0.5 cm using a medium soft (U40u) reconstruction kernel. In-plane resolution was 98 x 98 μm. Tumor volumetry was performed as stated above.

### Micro-CT in-phantom dosimetry

To determine the applied radiation dose during a micro-CT scan in-phantom method measurements were performed according to the AAPM TG-61 protocol for 40–300 kV X-ray beam dosimetry in radiotherapy and radiobiology [35]. The cylindrical air-filled ionization chamber PTW 31010 (PTW Freiburg GmbH, Freiburg, Germany) and a UNIDOS® E Universal Dosemeter (PTW Freiburg GmbH, Freiburg, Germany) were used. The chamber was placed at a conventionally used source surface distance (SSD) of 37 mm in the corresponding water-equivalent RW3 slab phantom 29672/U6 (PTW Freiburg GmbH, Freiburg, Germany).

### Clinical CT in-phantom dosimetry

Dose measurements were conducted under the same conditions used for in vivo tumor imaging of mice (scan length 10 cm). Dose measurements were performed with a vented pencil type PTW CT chamber calibrated on air kerma length product (chamber type: PTW-30009, energy range: 70–150 kV photons) and a UNIDOS® E Universal Dosemeter (both PTW Freiburg GmbH, Freiburg, Germany). The measuring volume of the CT chamber is 3.14 cm^3^ and the effective length is 100 mm. Daily temperature and pressure corrections were applied before irradiation.

As a linear measure of dose distribution over the full length of the CT chamber, the CT Dose Index was acquired; first free in air (CTDI_air_) [36], thereafter coated with a bolus of 1 cm thickness (water-equivalent material) in order to simulate the body of a mouse (CTDI_100_) [36].

### Partial brain irradiation

Three tumor bearing animals underwent radiation therapy using the micro-CT. For this purpose, the cone beam was collimated applying an in-house stainless steel tube to generate a pencil beam with a 5 mm bore [28]. Fractionated partial brain irradiation (3 x 5 Gy every other day) was performed from 3 different angels resulting in a total dose of 15 Gy.

### Histopathological analysis

After scanning on both CTs, all animals were sacrificed. Brains were removed and placed in 4% paraformaldehyde. For hematoxilin and eosin (H&E) staining the brain was sliced into coronal sections and the slides were used for tumor pathology and tumor volumetry. All histological slides were analyzed using an upright microscope combined with the digital microscope camera Leica DFC 450 (Leica Microsystems, Switzerland).

In each section with evident brain tumor pathology, the tumor area was outlined and calculated using ImageJ software (Version 1.44i., National Institut of Health, USA). Slide tumor volumes were calculated by multiplication of tumor area with the corresponding slice thickness. Brain tumor volumes were approximated by adding up the slide tumor volumes for each animal.

### Statistical analysis

As a measure of central tendency for continuous data means and standard deviations were used. Taking into consideration the small sample size and the observed distribution of the volumetric data, nonparametric statistics for related samples were used for hypothesis tests of group comparisons (Wilcoxon matched-pairs signed-rank test) and correlation analyses (Spearman’s rho). Since agreement between raters was excellent, imaging modality group comparisons were done for the first reader only. All statistical analyses were regarded exploratory with a significance level of p = 0.05. JMP® version 12 was used for statistical analysis.

### Measurement of the contrast-to-noise and signal-to-noise ratio

Mesurements were performed by one observer using OsiriX imaging software (OsiriX v. 5.0.2, 64-bit). To calculate the signal-to-noise ratio (SNR) and contrast-to-noise ratio (CNR) of all micro-CT and clinical CT scans, the mean CT number (attenuation) and mean SD were measured by placing a circular region of interest (ROI) in the the contrast enhancing tumor regions and in the surrounding tissue (brain parenchyma). The SNR for micro-CT and clinical CT was calculated separately by dividing the mean CT number in the tumor by the mean SD of the surrounding tissue (brain parenchyma). The CNR was calculated as follows: ROIa—ROIb/SDb, where ROIa is the mean attenuation coefficient of a defined structure in the region of interest (= tumor with contrast enhancment), ROIb is the mean attenuation coefficient of the image background sourrounding this structure (= contralateral brain hemisphere) and SDb (general background noise) is the SD of the CT numbers outside of the targeted region of interest (air-filled space outside the animal) [31, 37].

## Results

### Comparison of clinical CT imaging with micro-CT imaging and histology

**Fig 1:**
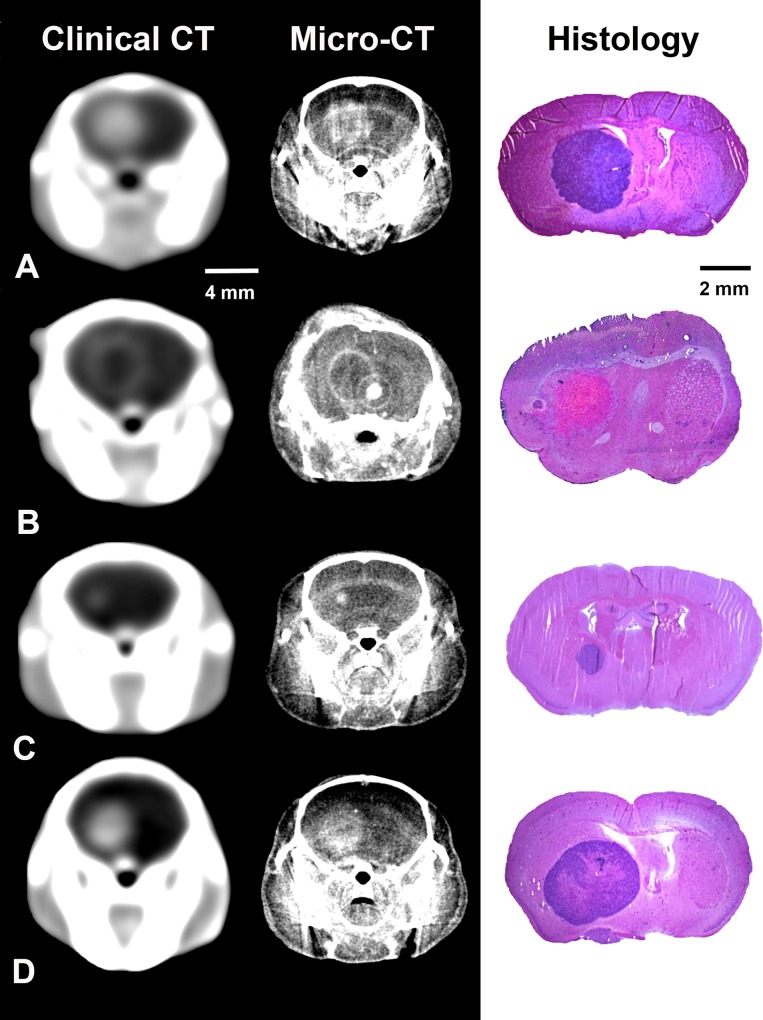
In vivo-images of orthotopically growing xenografts in the murine brain acquired using a clinical CT scanner (left row), a micro-CT scanner (middle row), and the corresponding histological sections (right row, H&E staining). Exemplary, tumors of different sizes and with different patterns of contrast agent uptake are shown. A) Large tumor with homogeneous contrast enhancement. B) Ring enhancement of another large tumor corresponding with central tumor necrosis. C) Small tumor. D) Middle-sized tumor with strong and homogeneous contrast enhancement.

Clinical and micro-CT scans were performed in nine mice and evaluated independently by two experienced readers. Histological analysis was performed in eight mice. The smallest tumor volume was 0.1 mm^3^ (as measured by histology) and clearly detectable in micro-CT as well as the clinical CT scanner. Animals (n = 3) treated by radiation therapy (3x5 Gy) showed ring-like contrast enhancement consistent with central tumor necrosis detectable in both, micro-CT and clinical CT. Exemplarily, Fig 1 illustrates orthotopically growing gliomas acquired by *in vivo* micro-CT, clinical CT and subsequent histological workup.

**Fig 2:**
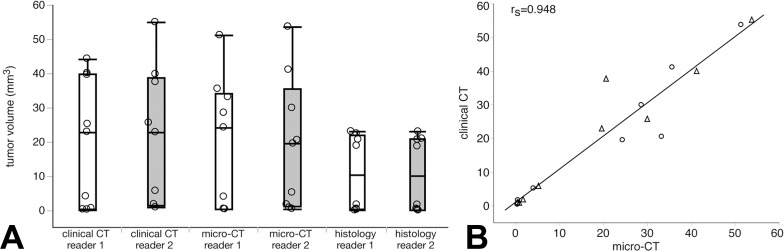
A) Tumor volumes measured by micro-CT, clinical CT, and histology for each reader separately. Within each modality, between reader volume analyses was similar (micro-CT p = 0.441, clinical CT p = 0.173, histology p = 0.161). Moreover, volume analysis was similar between micro-CT and clinical CT (reader 1: p = 0.813). Tumor volumes measured by histology were significantly smaller compared to both micro-CT (reader 1: p = 0.012) and clinical CT (reader 1: 0.012), respectively. Boxes indicate 25% and 75% quantiles, horizontal lines in boxes indicate median values, vertical bars indicate minimum and maximum, circles indicate measured tumor volumes. B) Correlation between tumor volumes as measured by clinical CT and micro-CT by two independent readers (circles for reader 1 and triangles for reader 2).

The mean±SD tumor volume quantified by reader 1 for clinical CT (n = 9), micro-CT (n = 9) and histology (n = 8) was 19.8±18.8, 19.8±19.0, and 11.0±11.2 mm^3^, respectively. Volumes determined from clinical CT and micro-CT images were statistically not different (p = 0.813). Histologically acquired tumor volumes were smaller due to shrinking artifacts compared to CT measurements (p<0.05 for matched-pair comparisons of histology versus clinical CT and histology versus micro-CT, respectively). Fig 2A depicts the volumetric measurements for all three modalities and both readers separately.

The within-modality measurements between readers showed excellent correlation for micro-CT (ρ/rs = 0.983, p<0.01), clinical CT (ρ/rs = 0.983, p<0.01), and histology (ρ /rs = 0.976, p<0.01). Likewise, the between-modality analyses of each reader exhibited excellent correlation for micro-CT and clinical CT (reader 1: 0.967, reader 2: 1.0, both p<0.01), micro-CT and histology (reader 1: 0.810, reader 2: 0.833, both p<0.05), and clinical CT and histology (reader 1: 0.833, reader 2: 0.833, both p<0.05).

### Radiation dose measurements

The in-air measurement yielded CTDI_air_ = 663.5mGy*cm/10cm = 66.4mGy point dose (scan length 10 cm). The measurement of the CT ionization chamber coated with 1 cm bolus yielded CTDI_100_ = 626.8 mGy*cm/10 cm = 62.7 mGy. For both measurements, the scan time was 87.22 s. For the latter measurement, the output data from the CT scanner provided a weighted CTDI_w_ result of 63 mGy and a dose length product of DLP = 728 mGy (DLP = CTDI_w_/pitch*scan length). Thus, our point dose measurements are in agreement with the dose values reported by the CT vendor (Siemens, Erlangen, Germany).

In-phantom dosimetry for the used micro-CT and scan protocol revealed a radiation dose of 0.48 Gy per scan at a depth of 3.2 mm (tube voltage of 80 kV, current 75 μA, 33 s scan time, 1,000 projections). For the used clinical CT scanner and scan protocol (tube voltage of 120 kVp, 150 mAs, 87 s scan time) a radiation dose of 0.094 Gy per scan was measured.

### Contrast-to-noise and signal-to-noise ratio measurements

The mean±SD CNR and SNR for micro-CT images were 2.1±1.0 and 1.1±0.04 and for images obtaind using a clinical CT scanner were 23.1±24.0 and 1.9±0.7.

## Discussion

Small animal models have become indispensable in preclinical neuro-oncologic studies [38–40]. Imaging became a highly relevant factor for monitoring treatment effects and thus therapeutic efficacy for both, clinical and experimental studies [41–44]. *In vivo* imaging of rodents has been challenging due to the small animal size and limited spatial resolution of imaging modalities [44]. Nowadays, several imaging techniques in clinical routine (e. g. CT, MRI, PET/SPECT) provide suitable resolutions for preclinical research in animals even as small as mice [24, 26]. Because of to the increasing relevance of medical imaging in translational research, dedicated small animal scanners with sufficient spatial resolution and signal-to-noise ratio are commercially available. However, such highly sophisticated equipment is not available to all institutions active in the field of experimental neuro-oncology due to high investment and maintenance costs [14, 45–48].

Micro-CTs in general provide high-resolution images (typically 50 μm or less) with varying acquisition times (20 s to 30 min), depending on the setup and device used [16, 49, 50]. Improvements in micro-CT imaging did not only allow to visualize brain vessels (for e.g. studies on stroke and vasospasm) [17, 22], but also to detect brain tumors in animals as small as mice [6, 51]. Compared to micro-CT scanners, the use of clinical CT scanners for brain tumor imaging in mice has been considered critical due to the limited spatial resolution. Accordingly, the verification of glioma growth in living mice using a clinical CT scanner has been reported in only one study [46]. To the best of our knowledge the applicability of clinical CT scanners for *in vivo* volumetric analyses and for the visualization of morphologic brain tumor changes in mice has not been investigated yet.

In this study we were able to show, that (I) even small brain tumors are accurately detectable in images acquired using a clinical CT scanner. (II) More importantly, volumetric analyses revealed an overall excellent correlation between clinical CT and micro-CT (Fig 2B). (III) Furthermore, we were able to show that treatment effects like central necrosis can reliably be detected using a clinical CT scanner (Fig 1B).

In addition to the high availability, benefits of using clinical CT scanners are comparatively fast scan protocols, low effort of data acquisition and a relatively large field of view, which allows for scanning several mice simultaneously. The fast acquisition times of clinical CT scanners are also beneficial with respect to the application of contrast agent. As some micro-CT scanners have comparably long scan times, it may be necessary to continuously infuse contrast agent during a scan[52, 53]. Furthermore, shorter scan times make anaesthesia of the animals easier and less physically demanding. Thus, in recent studies ultrafast scan protocols (20–40 s) were established for high-resolution micro-CT of living mice [17, 19, 22, 54].

An important factor frequently discussed in the context of X-ray imaging are radiation doses applied during serial scanning. Although radiation exposure in micro-CT is far from lethal, the applied doses have been discussed to be sufficient to bias (or confound) experimental outcomes [29, 55]. Thus, X-ray based imaging has been reported to influence oncological studies, especially in longitudinal studies with repetitive CT scans [16, 50, 56, 57]. In our study radiation doses of approximately 0.1 Gy per scan were measured using the clinical scanner, which is 5 times lower than the doses measured for our standard micro-CT protocol (approx. 0.5 Gy per scan). Previous studies have suggested that mouse imaging using micro-CT applies between 100 and 300 mGy, which is about 10 times more than expected from a clinical CT scan used for human diagnostics [15, 49]. The relatively high radiation doses of micro-CT in our setting were mainly caused by minimization of the source-object-distance (inverse-square law), which we applied to maximize spatial resolution. At this point it is important to mention, that it would have been possible to reduce radiation exposure for micro-CT scans in our study by reducing e. g. the source-object-distance (SOD), acquisition time, number of projections, x-ray tube current (mAs) and tube voltage (kV). We did not perform controlled experiments to investigate the influence of dose reduction in micro-CT on image quality (incl. SNR), which would be affected negatively by dose reduction [58–60].

The SNR and CNR values measured in our study have to be interpreted with care. Values obtained for micro-CT and clinical CT cannot be easily compared, as they depend on several factors. These include slice thickness, reconstruction kernel, reconstruction algorithm, SOD, acquisition time, X-ray tube current (mAs) and tube voltage (kV).

A general limitation of using a clinical CT scanner is the limited spatial resolution, which might be insufficient to detect subtle changes in tumor volume and tumor morphology for studies evaluating treatment response. On the other hand we were able to detect very small as well as large tumors, and we even observed treatment related changes in tumor morphology like necrotic areas. With respect to very small changes in tumor volume however, the clinical relevance might become debatable.

In most cases we observed a trend towards lower tumor volumes in histologic slides compared to CT image analysis. During fixation, tissues commonly change in volume. Shrinkage is almost unavoidable and thereby introducing possibly the most significant artifact of a histological procedure. Tissues fixed in formaldehyde and embedded in paraffin wax have been reported to shrink up to 33% [61], whereas other studies reported even stronger shrinkage when tissues were subjected to fixation, cryoprotection, or embedding routines [62, 63]. Furthermore, some tumors exhibited large central necrotic areas and impression was that these necrotic tumors were even more prone to shrinkage artifacts in our dataset.

The majority of imaging experiments make use of immune deficient animals, primarily severe combined immune deficient (SCID) and nude mice. To ensure the health of these animals over the course of imaging experiments (which can last several weeks) a pathogen barrier around the animals must be maintained around at all times. In our study the clinical CT scanner was located outside of the barrier facility. To maintain the specific pathogen free (SPF) status of our animal facility, mice were not allowed to be returned into the barrier after being examined in the clinical CT scanner. Therefore, a longitudinal tumor evaluation was not possible with the clinical CT scanner, i.e. only a final scan could be performed. In different settings however, a longitudinal analysis might be feasible.

In a former study [6] we investigated the applicability of BLI to determine tumor size of untreated and irradiated orthotopic glioblastoma xenografts in mice. Corresponding to other studies [51] BLI presented with a high sensitivity and perfect specificity. Whereas the correlation between tumor volume and BLI signal was significant, the strength of the correlation was only moderate. This is in accordance with other studies [4] and especially applies to the volumetric extremes of very small and large tumors, respectively.

In conclusion, we demonstrated that clinical CT scanners may reliably be used for *in vivo* imaging and volumetric analyses of brain tumor growth in mice. Moreover, clinical CT scanners allow the *in vivo* detection of macroscopic changes of tumor morphology in mice. Thus, clinical CT scanners may be used for preclinical neuro-oncologic imaging of mice.

## Supporting Information

S1 Fig Contrast enhanced micro-CT and corresponding histology of a mouse carrying a brain tumor induced by intracerebral injection of primary tumor cells. Note the non-enhancing tumor causing a midline shift.
(TIF)

S1 File Data file underlying the results presented in the manuscript. (XLS)
