# Supplementary figures and images for: Imaging of Orthotopic Glioblastoma Xenografts in Mice Using a Clinical CT Scanner: Comparison with Micro-CT and Histology
Source: PLoS One. 2016 Nov 9;11(11):e0165994. doi: 10.1371/journal.pone.0165994 (PMC5102379; doi:10.1371/journal.pone.0165994)

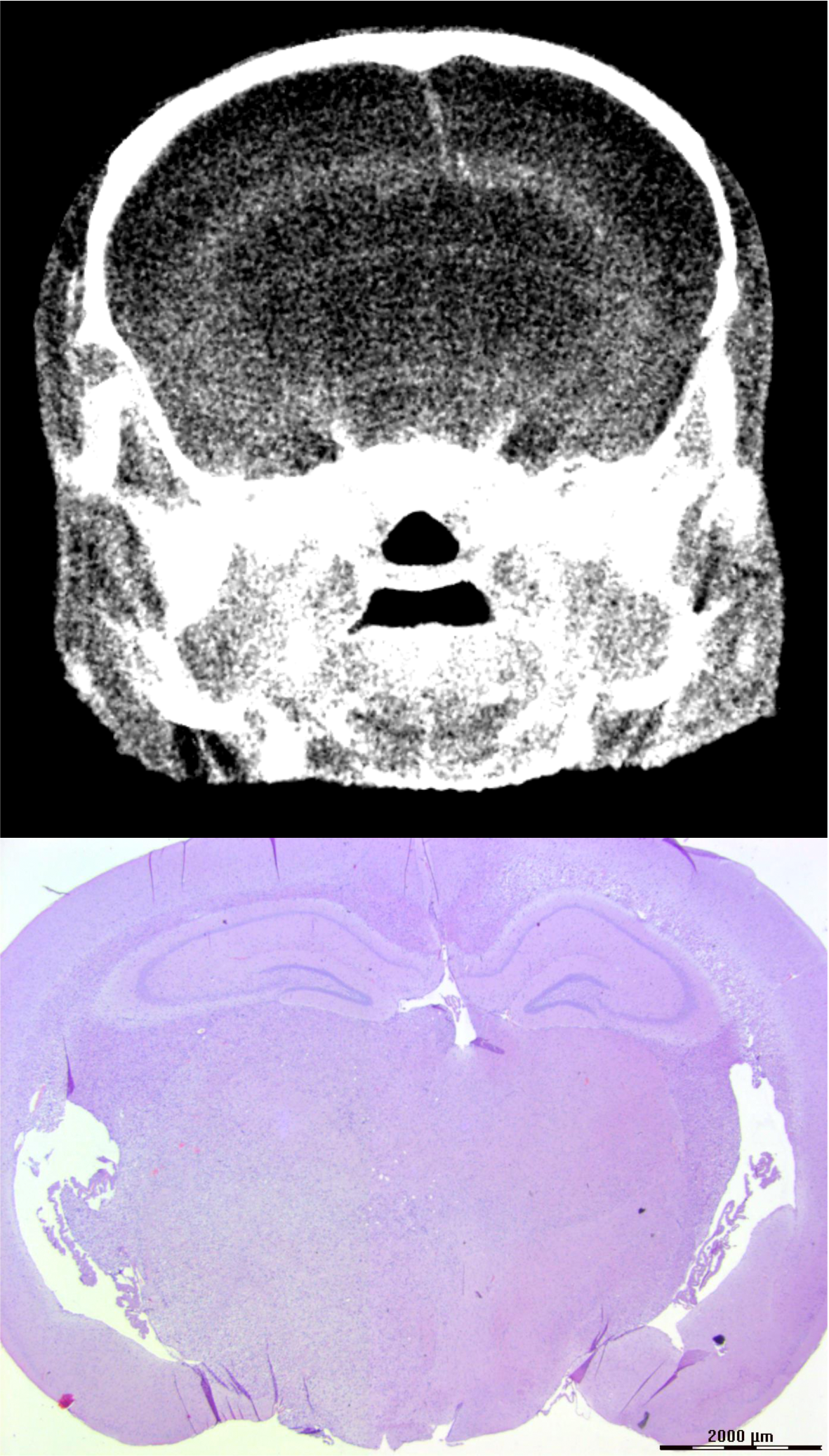

Supplement: S1 Fig — Note the non-enhancing tumor causing a midline shift. (TIF) [file pone.0165994.s001.tif]
